# Role of external stimulation in shaping evoked activity in a macroscopic model of cortex

**DOI:** 10.1186/1471-2202-15-S1-P175

**Published:** 2014-07-21

**Authors:** Matthieu Gilson, Adrian Ponce-Alvarez, Gustavo Deco

**Affiliations:** 1 Dept. de Tecnologies de la Informació i les Comunicacions, Universitat Pompeu Fabra, Barcelona 08018, Spain

## 

The activity of the brain exhibits stereotypical patterns at various temporal and spatial scales. Novel techniques such as functional magnetic resonance imaging (fMRI) allow the simultaneous recording of the macroscopic activity from all brain regions while subjects perform various tasks, such as passive hearing and active visual recognition. How neural activity is related to cognitive tasks is a long-standing question for the neuroscience community. This study examines an interesting aspect of this broad question, namely the comparison between the resting state (when the subjects is idle) and evoked activity during a task. Neural activity patterns consist of the correlations of firing activity between cortical regions - or functional connectivity - at a slow time scale (hundreds of ms), which can be related to data obtained from fMRI.

The cortex has an intricate architecture of connections, both local and long-range. The latter have been mapped for the whole human cortex using diffusion techniques [1]. A recent model has demonstrated that this broad-scale structural connectivity is crucial to reproduce resting-state patterns of cortical activity [2]. Here we consider the same model and simulate tasks by exciting specific brain regions. We rely on mathematical and numerical analysis in order to uncover the respective roles of the long-range projections and external inputs in shaping the evoked activity. Our primary aim is to understand how the stimulation of specific brain regions can dramatically modify the functional connectivity, as compared to the resting state. We also examine the influence of long-range excitatory projections onto inhibitory populations, a novel dynamical ingredient compared to the previous model, in decorrelating brain regions.
